# Sources and Specified Health Risks of 12 PM_2.5_-Bound Metals in a Typical Air-Polluted City in Northern China during the 13th Five-Year Plan

**DOI:** 10.3390/toxics12080581

**Published:** 2024-08-10

**Authors:** Deai Yang, Mingjun Li, Xingyi Geng, Zhihui Feng

**Affiliations:** 1 Department of Labor Hygiene and Environmental Hygiene, School of Public Health, Cheeloo College of Medicine, Shandong University, Jinan 250012, China; 202236505@mail.sdu.edu.cn; 2 Jinan Municipal Center for Disease Control and Prevention Affiliated to Shandong University, Jinan 250021, China; lmjcheer@163.com

**Keywords:** PM_2.5_-bound metals, PMF, source, health risk, emission reduction, 13th Five-Year Plan

## Abstract

The continuous monitoring of PM_2.5_ (including 12 metal elements) was conducted in Jinan, a city with poor air quality in China, during the 13th Five-Year Plan (2016–2020). Positive matrix factorization (PMF) was used to identify emission sources of PM_2.5_-bound metals, and the health risks of the metals and their emission sources were assessed. During the study period, the concentration of most metals showed a decreasing trend (except Al and Be), and a significant seasonal difference was found: winter > fall > spring > summer. The PMF analysis showed that there were four main sources of PM_2.5_-bound metals, and their contributions to the total metals (TMs) were dust emissions (54.3%), coal combustion and industrial emissions (22.3%), vehicle emissions (19.3%), and domestic emissions (4.1%). The results of the health risk assessment indicated that the carcinogenic risk of metals (Cr and As) exceeded the acceptable level (1 × 10^−6^), which was of concern. Under the influence of emission reduction measures, the contribution of emission sources to health risks changes dynamically, and the emission sources that contribute more to health risks were coal combustion and industrial emissions, as well as vehicle emissions. In addition, our findings suggest that a series of emission reduction measures effectively reduced the health risk from emission sources of PM_2.5_-bound metals.

## 1. Introduction

In recent years, with accelerating urbanization and ecological degradation, air pollution, mainly particulate matter (PM), has become increasingly frequent and severe, raising global concerns about its health impacts, particularly in developing countries [1]. PM with an aerodynamic diameter of less than 2.5 μm is the most concerning air pollutant, with studies indicating that 86% of the global urban population in 2019 resided in areas exceeding PM_2.5_ exposure limits. This led to 1.8 million deaths, including 852,000 in China, attributed to atmospheric PM_2.5_ pollution [2,3]. PM_2.5_ exerts detrimental health effects at the molecular level through various toxic mechanisms, including oxidative stress, mitochondrial damage, immune dysregulation, DNA damage, etc., highlighting its extensive impact on human health [4,5,6]. For instance, PM_2.5_ can induce oxidative stress through the catalysis of biochemical reactions, the activation of oxidative and metabolic enzymes, and mitochondrial dysfunction, exacerbating various respiratory diseases [7]. The large specific surface area of PM_2.5_ enables it to adsorb numerous toxic substances, including toxic metals, polycyclic aromatic hydrocarbons (PAHs), water-soluble ions, and carbonaceous compounds. Although metal elements account for a small proportion of PM_2.5_, the health risks they pose cannot be ignored. Generally, the metals that humans encounter most come from foods. For instance, Hg and its methylated forms in aquatic products can cause damage to the nervous system and kidneys, and lead to immune system suppression [8], while airborne toxic metals primarily enter the human body through the respiratory tract, reaching the bloodstream via the alveoli and accumulating over time. Studies have linked long-term exposure to metal-rich PM_2.5_ (e.g., As, Cr, Cd, Ni, and Pb) to an increased risk of cancer and chronic adverse effects on the respiratory, cardiovascular, and nervous systems [9,10,11,12]. Children are particularly vulnerable due to their developing bodies, lower body weight, higher heavy metal absorption rates in the digestive system, and increased hemoglobin sensitivity to these metals [13]. Notable health detriments, encompassing cancer, contribute significantly to the mounting financial strain on national healthcare expenditures. Concurrently, the surge in cancer cases may exert forgotten impact effects on the environment, healthcare professionals, and the delivery of health services—a concern that is magnified by the ongoing increase in the aging population [14]. Hence, the detrimental impacts of PM_2.5_ associated with toxic metals represent a grave concern for public health. 

With the increasing demand for energy and industrial development, atmospheric emissions of metals are far exceeding background values, especially in areas of China with high levels of industrial and transportation activity [15]. Anthropogenic activities are the main source of metals in PM_2.5_, including industrial, coal combustion, motor vehicle, and dust emissions [16,17]. Differences in the type and contribution of pollution sources are due to the urbanization of the region and differences in energy structure. For example, source analysis of metals in PM_2.5_ in Saint-Omer, a medium-sized city in northern France, identified glass manufacturing, iron and steel smelting, and fuel combustion as the primary sources [18]. In contrast, toxic metals in PM_2.5_ in a typical industrial city in China originate mainly from industrial sources, motorized vehicle sources, mixed combustion sources, and dust sources [19]. The emergence of various air pollutant models, including air quality models, photochemical models, and receptor models, plays a significant role in understanding the health outcomes of populations in polluted areas as well as emission control [20]. Among them, receptor models excel in identifying the sources of pollutants, such as Chemical Mass Balance (CMB) [21], Principal Component Analysis (PCA) [22], and Positive Matrix Factorization (PMF) [23]. Of these, the PMF model is particularly effective because it avoids negative solutions and has been used successfully in published studies for the quantitative source identification of PM_2.5_-bound metals [24,25]. By understanding the sources of metallic elements, steps can be taken to minimize their presence in PM and further reduce health risks.

As the capital of Shandong Province, Jinan has been reported by the World Health Organization (WHO) as one of the most polluted cities worldwide due to its basin topography, industrial structure, and the proliferation of vehicles, which have led to the accumulation of atmospheric pollutants over the city, presenting serious compound pollution [26,27]. Despite continued declines in PM_2.5_ concentrations with the Jinan government’s refined management of air pollution prevention and control, levels still far exceed the WHO standard (35 μg/m^3^). This severe pollution not only affects local economic growth but also adversely affects human health. However, few studies have investigated the health risks and sources of metals in PM_2.5_ in the region. Most published studies are based on short-term monitoring data, limiting the understanding of emission sources of metals in PM_2.5_ and the long-term characterization of their health risks [28,29,30]. Additionally, few studies have evaluated the impact of emission reduction policies on metals in PM_2.5_. In response to these limitations, this study aims to conduct a comprehensive analysis of metals in PM_2.5_ in Jinan during the 13th Five-Year Plan period (2016–2020) to explore the temporal characteristics, sources of pollution, and trends in health risks; to assess the effects of abatement measures during this period; and to provide data support for the optimization of atmospheric heavy metal pollution prevention and control measures in Jinan.

## 2. Materials and Methods

### 2.1. Sampling Sites and Methods 

**Figure 1:**
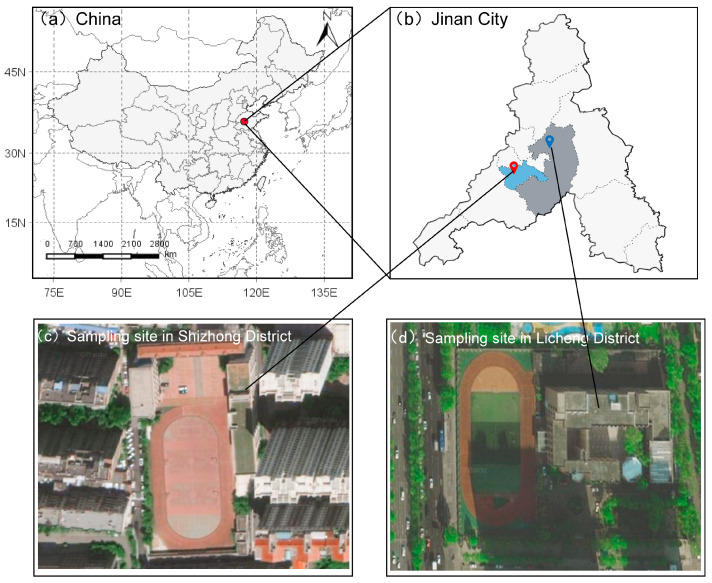
Geographical information of sampling sites: (**a**) map of China; (**b**) outline of Jinan City; (**c**) sampling site in Shizhong District; (**d**) sampling site in Licheng District.

As shown in Figure 1, Jinan is located in the east of China, belonging to the Beijing–Tianjin Wing and the Bohai Rim Economic Radiation Zone, and has a population of over 9 million people (in 2020). Drawing on air quality data from Jinan’s various districts prior to 2015, we identified Shizhong District (36.10° N, 117.00° E) as an area of comparatively better air quality and Licheng District (36.74° N, 117.15° E) as an area with comparatively poorer air quality for the sampling sites. Licheng District, an old industrial zone, is encircled by a multitude of heavily polluting enterprises, including thermal power plants, steelworks, and refineries. Conversely, Shizhong District is characterized by its bustling commercial and residential sectors with significant traffic activity. Additionally, the energy profiles and industrial distributions in these districts align well with the prevailing pollution emission patterns in the region, which consist of primarily industrial, vehicular, and coal-burning emissions. Consequently, the selection of Licheng and Shizhong districts for the sampling of PM_2.5_ and its metallic constituents aptly encapsulates the general air quality conditions of Jinan.

The sampling equipment was placed on the roof of the teaching building, and the sampling period was from January 2016 to December 2020, with seven consecutive days of routine monitoring from the 10th to the 16th of each month, sampling for 22–24 h per day, and with parallel samples and blank samples measured once a month. An ambient particulate sampler (zr-3920c, Zhongrui, Qingdao, China) was used to collect fine atmospheric particulate samples. During the investigative process, no metal contaminants that could potentially interfere with detection were found in either the filtering membranes or the field blank samples. To ensure minimal background contamination during sampling, the filtering membranes were pre-treated by heating at a temperature of 400 °C for a duration of 5 h within a muffle furnace. Both pre- and post-sampling, the filters were conditioned in a humidity-controlled chamber, set to a temperature of 20 ± 1 °C and a relative humidity of 50 ± 3%, for a duration exceeding 24 h and weighed using an automated filter weighing system (WZZ-E, Weizhizhao, Hangzhou, China). The sampling flow rate was set at 100 L/min, and the sampling filter was a quartz fiber filter (90 mm diameter, Whatman, Mettlestone, UK). All samples were sealed in clean plastic bags and stored in a freezer at −20 °C until further analysis. Concurrently, a comprehensive set of meteorological parameters was meticulously recorded throughout this timeframe, including temperature, relative humidity, wind speed, and wind direction.

### 2.2. Sample Treatment

The mass of the PM_2.5_ samples was ascertained by measuring the filter membranes’ weight both prior to and subsequent to the sampling process. Post weighing, the membranes were cut using ceramic scissors to ensure uniformity. Subsequently, each piece of the shredded filter membrane was transferred into a 15 mL screw-cap plastic vial (BD Falcon, Becton, Dickinson and Company, Bergen, NJ, USA), and 10 mL of 5% nitric acid (HNO_3_) was added. The tube was then sealed and immersed in a 70 °C water bath for 3 h of ultrasonic extraction in a fume hood. Afterward, the tubes were centrifuged at 4500 rpm for 5 min using a low-speed centrifuge (Centrifuge 5804, Eppendorf, Hamburg, Germany), and the supernatant was collected for analysis. The concentrations of 12 elements (Al, Hg, Mn, Sb, Se, As, Be, Cr, Cd, Ni, Pb, and Tl) were quantified in the digested supernatant fluid using an inductively coupled plasma mass spectrometer (ICP-MS; Model ICAP-QC, Thermo Fisher Scientific, Middlesex County, NJ, USA). To optimize the instrument’s sensitivity, oxide, double charge, and resolvability, the ICP-MS tuning solution (Li, Bi, Ce, Co, In, Ba, U at 1.0 μg/L; Thermo Fisher Scientific, Middlesex County, NJ, USA) was used. Standard curves were constructed using an instrument workstation. Quality assurance and control were performed using certified reference materials (CRMs; National Research Center for Certified Reference Materials, China), reagent blanks, and spiked recovery checks.

### 2.3. Positive Matrix Factorization (PMF) Model

The positive matrix factorization (PMF) model (version 5.0) is a receptor model that can identify different sources according to their characteristic components and quantify their contribution to pollutants [31,32]. The PMF model first calculates the error of each chemical component in the particulate matter by using the weight, and then determines the primary pollution source and its contribution rate to the particulate matter using the least squares method. A speciated data set can be viewed as a data matrix X of n by m dimensions, in which n number of samples and m chemical species are measured, as shown in Equations (1) and (2).

(1) $$e_{\mathrm{ij}}=X_{\mathrm{ij}}-\sum_{k=1}^{p}f_{\mathrm{kj}}g_{\mathrm{ik}}$$

(2) $$Q=\sum_{j=1}^{m}\sum_{i=1}^{n}{\left[\frac{e_{\mathrm{ij}}}{s_{\mathrm{ij}}}\right]}^{2}$$

where $e_{\mathrm{ij}}$ is the residual for each sample/species, $X_{\mathrm{ij}}$ is the measured concentration (in µg/m^3^), $s_{\mathrm{ij}}$ is the estimated uncertainty (in µg/m^3^), n is the number of samples, m is the number of species, and p is the number of sources included in the analysis. Q is used to characterize the size of the overall fitting residual after uncertainty conversion.

Following the PMF model’s criteria, the signal-to-noise (S/N) ratio serves as a metric for species classification [33]. A “Strong” classification is applied to species with an S/N ratio greater than 1. For ratios between 0.5 and 1, the species is deemed “Weak”. Conversely, an S/N ratio below 0.5 results in a “Bad” categorization. In this research, an exploration was conducted to evaluate between 3 and 6 potential factors, with the aim of identifying the most optimal number based on the Q value metric. During each iteration of the analysis, the stability and reliability of the results were rigorously assessed. This assessment was based on several criteria, including the Q value, a detailed residual analysis, and the correlation coefficient that compared the observed concentrations with those predicted by the model. The details of the PMF process are provided in the Supplementary Materials (Section S1).

### 2.4. Health Risk Assessment

Non-carcinogenic (Mn, As, Cd, Cr, Al, Ni, Sb, Se, Be, and Hg) and carcinogenic (Cr, As, Cd, Pb, Ni, and Be) risk assessments were conducted for the 11 metal elements according to the U.S. EPA’s Integrated Risk Information Database and the IARC [34,35]. The average daily dose (ADD) and lifetime average daily dose (LADD) by inhalation route were calculated using Equations (3) and (4):

(3) $$\mathrm{ADD}=\frac{C\times \mathrm{IR}\times \mathrm{EF}\times \mathrm{ED}}{\mathrm{BW}\times \mathrm{AT}}$$

(4) $$\mathrm{LADD}=\frac{C\times \mathrm{EF}}{\mathrm{LT}}\times \left(\frac{{\mathrm{IR}}_{\mathrm{child}}{\mathrm{ED}}_{\mathrm{child}}}{{\mathrm{BW}}_{\mathrm{child}}}+\frac{{\mathrm{IR}}_{\mathrm{adult}}{\mathrm{ED}}_{\mathrm{adult}}}{{\mathrm{BW}}_{\mathrm{adult}}}\right)$$

A detailed description of the parameters is given in Table S1. The concentration of Cr(VI) was estimated to be one-seventh of the total Cr, as studies have indicated that the ratio of Cr(VI) to Cr(III) is approximately 1:6 [36]. Given that exposure parameters such as IR, BW, and ED are age-dependent, the ADD for a substance can differ significantly across various age groups [37]. LADD was estimated through the time weighting of exposures occurring in childhood and adulthood. 

The hazard quotient (HQ) and carcinogenic risk (CR) through the inhalation route can be calculated using Equations (5) and (6), and the toxicological parameters of the metals are shown in Table S2.

(5) $$\mathrm{HQ}=\frac{\mathrm{ADD}}{\mathrm{RfC}}$$

(6) $$\mathrm{CR}=\mathrm{LADD}\times \mathrm{IUR}\times 10^{3}$$

The hazard index (HI) is the sum of HQ for all metals; HQ or HI > 1 indicates a high probability of occurrence of adverse non-carcinogenic effects over a lifetime. The CR is quantified as the likelihood of an individual developing cancer due to a lifetime of exposure to carcinogenic substances. The benchmark for a tolerable threshold of cancer risk is set at 1 × 10^−6^, signifying that, under this risk level, there is an expectation that one individual among a million might contract cancer from continuous, lifelong exposure to hazards.

### 2.5. RM-RA Model

By combining the receptor model with the risk assessment model, a health risk assessment method for specific pollution sources was established to calculate the harm of different emission sources on health risks. The equation used is as follows:

(7) $$C_{\mathrm{ij}}^{k}=g_{\mathrm{ij}}\times f_{\mathrm{ij}}$$

$C_{\mathrm{ij}}^{k}$ is the mass concentration of metal element j contained in source k in sample i (unit: µg/m^3^), and the calculated $C_{\mathrm{ij}}^{k}$ should be used in Equations (3)–(6) to estimate the carcinogenic and non-carcinogenic risks of the metal elements in each pollution source.

## 3. Results and Discussion

### 3.1. Long-Term Concentration Trends in PM_2.5_-Bound Metals

**Figure 2:**
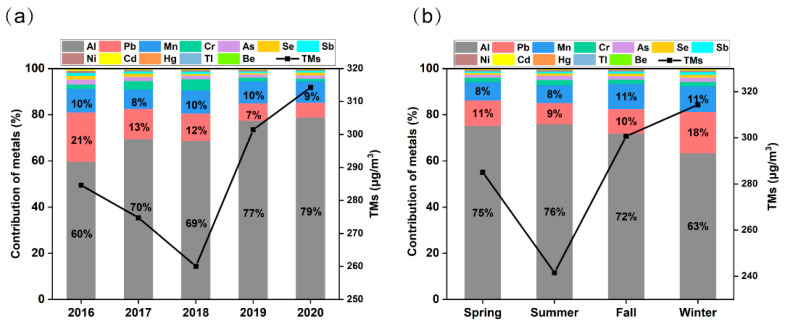
The concentrations’ TMs (broken line) and the contribution of each metal to TMs (%, stacked histogram): (**a**) annual trends; (**b**) seasonal differences.

Preliminary analysis of the sampling data revealed that the concentrations of PM_2.5_-bound metals in the two sampling areas showed the same trend over the study period, and significant correlations were found between the same metals (Figures S1 and S2). Therefore, we consider that the average concentrations of pollutants in the two monitoring areas can well represent the pollution situation in the urban area of Jinan. Among the 12 PM_2.5_-bound metals, the concentrations of most metals (except Al and Be) have declined obviously in Jinan since 2016 (Figure 2a), while the concentrations of total metals (TMs) increased by 10%, from 284.6 ng/m^3^ (in 2016) to 314.2 ng/m^3^ (in 2020), due to a significant increase in Al concentrations. The top three metals with substantial proportions were Al (54–1250 ng/m^3^), Pb (5–247 ng/m^3^), and Mn (5–121 ng/m^3^), accounting for 92% of the TMs. The remaining 8% comprised Sb, As, Be, Cd, Cr, Hg, Ni, Se, and Tl, each of which was less than 10 ng/m^3^ (Table S3). Between 2016 and 2020, Pb experienced the most significant decrease in concentration, with a substantial reduction of 70%, followed by Tl (68%), Cd (61%), Sb (41%), Ni (41%), As (33%), Cr (32%), Se (26%), and Mn (6%). However, there was an increase in the concentrations of Al and Be, which were 36% and 29%, respectively. The continuous increases in concentrations of Al and Be were attributed to the increase in building and road construction activities in Jinan in recent years, and previous studies have shown that Al and Be were mainly caused by to dust emissions, according to source apportionment [38,39]. 

As shown in Figure 2b, significant seasonal differences were observed in the TMs and proportions of PM_2.5_-bound metals. Generally, the concentrations of most metals (except Al and Be) were higher in winter and lower in summer. These seasonal variations were mainly driven by the meteorological conditions in Jinan: a lower planetary boundary layer (PBL) occurs in fall and winter, and scarce precipitation and high pressure are not conducive to air self-purification, while higher PBL, abundant precipitation, low pressure, and better air circulation occur in spring and summer [40,41]. In this research, atmospheric pressure and temperature significantly influenced the concentration of metals (Figure S3). The interplay between these elements and human activities has amplified pollution levels. For example, the substantial combustion of fossil fuels like coal for heating in winter in the area results in the emission of related metals. Additionally, the high atmospheric pressure and low temperatures characteristic of the winter impede the dispersion of pollutants through atmospheric circulation, contributing to increased regional pollution [42]. Overall, meteorological factors in Jinan indirectly impact the pollution effect, whereas anthropogenic activities are still mainly attributed as the source of air pollutants [43].

### 3.2. Characterization of Emission Sources of PM_2.5_-Bound Metals

#### 3.2.1. Emission Sources Apportioned by PMF Model

This study conducted source apportionment analyses to decipher the origins of PM_2.5_-bound metals. The optimal setting scheme was determined by analyzing the distribution of factors, Q-values, and differences between the actual and predicted values for different metals [44]. When three factors were identified, the tracer elements of vehicular and industrial emissions were difficult to distinguish, whereas when the number of factors exceeded three, the characteristic elements of vehicular and industrial factors were well separated (Figure S4). The results indicate that four factors are considered optimal solutions.

As depicted in Figure S5, Factor 1 is predominantly composed of Mn (87.2%), Cr (65.3%), Sb (59.4%), and Ni (51.1%), and these metals are identified as the characteristic components of vehicle emissions. Mn and Ni are usually used as indicators of gasoline or diesel combustion [32,45,46], and the high contents of Cr and Sb may be related to the wear of brake pads and tires, as well as rust particles from vehicles [47,48]; therefore, Factor 1 is named vehicle emissions.

Factor 2 mainly comprises Al (79.2%) and Be (38.3%). It has been indicated that Al comes from soil dust, a typical crustal element, and the main sources of Be are various ores [49,50]. In urban areas, the sources of Al and Be in the air are mainly road and construction dust, while frequent dust weather in fall and winter increases their concentrations simultaneously [51]. Thus, Factor 2 is collectively referred to as dust emissions. 

Factor 3 has high Pb (85.5%), Cd (83.0%), Se (42.2%), and As (25.2%) loads. Pb and Cd are generally considered common metal emissions in the metallurgical industry, while studies have shown that Se and As are characteristic elements in coal combustion processes, such as smelting, thermal power generation, and heating [52,53,54,55]. Therefore, factor 3 is identified as a mixed source of coal combustion and industrial emissions.

Most of the metal elements in Factor 4 are generally underrepresented (<20.0%), except for As (72.3%), which previous studies have shown to be derived primarily from the combustion of fossil fuels [56]. It is understood that many households in the region still use coal and biomass for cooking, which can also contribute to the increase in concentrations of PM_2.5_ and metals [57,58]. Thus, Factor 4 can be identified as domestic emissions.

#### 3.2.2. Annual Trends in Sources: Concentrations and Contributions

**Figure 3:**
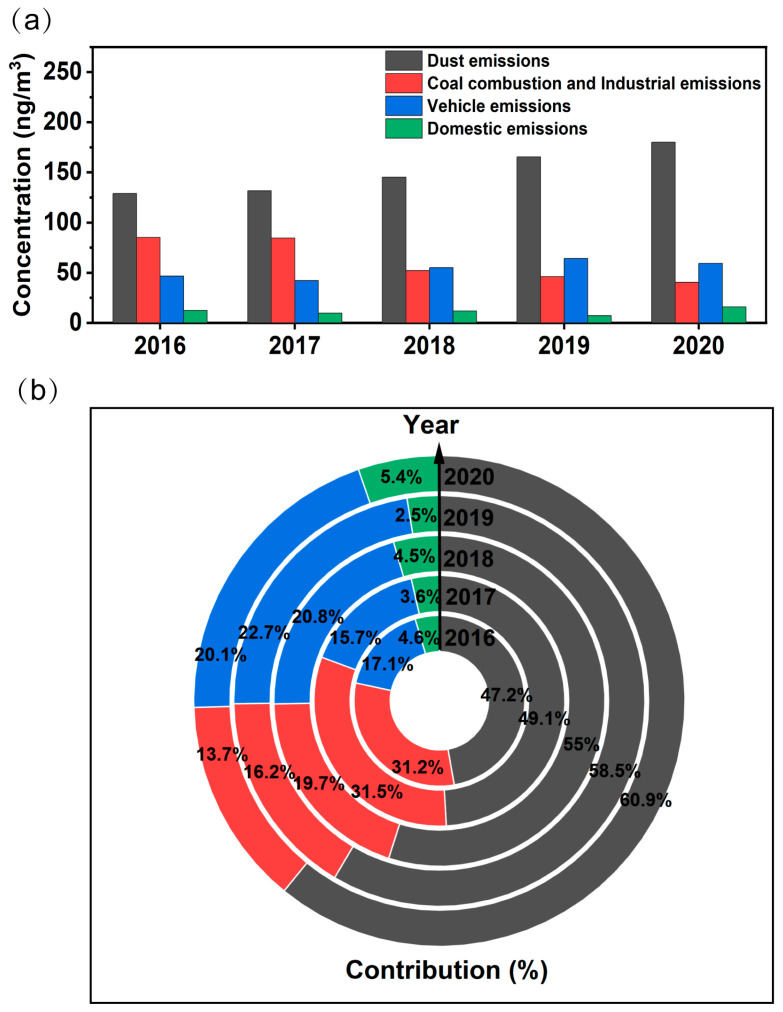
The characteristics of sources from 2016 to 2020: (**a**) concentrations and (**b**) contributions to TMs.

Figure 3a showed the concentrations and contributions from each source to TMs between 2016 and 2020. Metals from dust emissions maintained high concentrations mainly due to the high proportion of Al, and this was similar to the results of a previous study [59]. Metals from coal combustion and industrial emissions gradually declined under effective control measures (such as the relocation of highly polluting enterprises and the promotion of clean energy). In contrast, the concentration of vehicle emissions has been on the rise in recent years owing to the number of vehicles. Concentrations of domestic emissions were the lowest compared to those of other sources, and as civic literacy improved, people were paying more attention to environmentally friendly lifestyles.

During the study period, the average contributions of dust and industrial, vehicle, and domestic emissions to the concentration of TMs were 54.3%, 22.3%, 19.3%, and 4.1%, respectively, while the contributions of sources varied under the influence of emission reduction strategies in different periods. Urbanization in Jinan has gradually accelerated in recent years, reflected in the expansion of building construction and road paving areas as well as severe weather (such as sandstorms), all of which have led to the dominance of dust emissions [60]. Interestingly, the contribution of vehicle emissions has exceeded that of coal combustion and industrial emissions since 2018, and this has been attributed to the government’s stringent controls on industry and suggests that the focus of air pollution control should shift to vehicle emissions.

### 3.3. Health Risk Assessment

#### 3.3.1. Non-Carcinogenic and Carcinogenic Risks of Metals of Concern: Mn, As, and Cr

**Figure 4:**
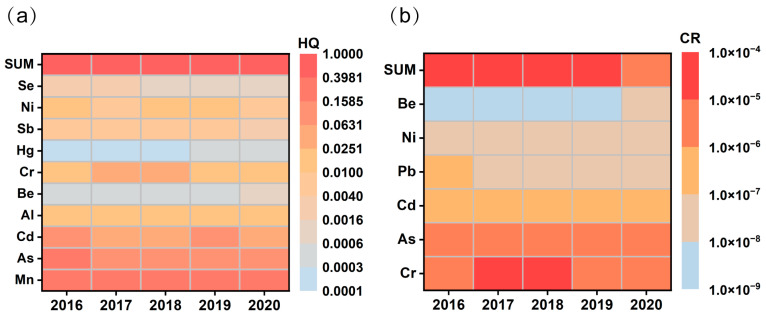
Health risk assessment of PM_2.5_-bound metals during 2016–2020: (**a**) HQ and (**b**) CR of different metals (darker grids represent higher HQ or CR values).

As shown in Figure 4a and Table S4, children exhibited an average HI of 0.60 for metal exposure, while adults demonstrated a lower average HI of 0.37 during 2016–2020. Both of these averages fall beneath the critical threshold of 1. Exposure parameters (e.g., IR and BW) differed significantly between children and adults, resulting in a higher HI in children than in adults [61]. The ranking of the HQ of metals for adults and children was Mn > As > Cd > Cr > Al > Ni > Sb > Se > Be > Hg. Among them, the contribution of Mn to the average HI in adults and children was 51.0%, of As was 24.9%, and of Cd was 12.3%, and other metals’ contributions ranged from 0.1% to 5.2%. This result was consistent with prior research highlighting the elevated HQ of Mn in urban particulate matter in China [62,63]. A clear downward trajectory characterizes the HI from 2016 to 2020, with an overall decline of 26.5%. The reduction in the HQ for each metal is in direct correspondence with the decrease in its concentration. Cd demonstrated the most pronounced decrease in HQ, with a 61.2% reduction during the study period. This was followed by significant declines for Sb at 41.3% and Ni at 40.1%. Although Mn had the highest HQ among the metals initially, its reduction was the least substantial, at only 5.5%. Therefore, the continued monitoring and mitigation of Mn in Jinan is of critical importance. 

As shown in Figure 4b and Table S5, between 2016 and 2020, the average total CR (TCR) for adults and children was 1.4 × 10^−5^. The risk level of metals was Cr > As > Cd > Pb > Ni > Be, of which Cr contributed 70% of the TCR. Although the TCR decreased by 35.1% from 1.7 × 10^−5^ (in 2016) to 1.1 × 10^−5^ (in 2020), it still exceeded the threshold of CR (1.0 × 10^−6^). The CR of Cr exhibited a relatively modest reduction of 32% between 2016 and 2020. Nonetheless, its value in 2020, at 5.7 × 10^−6^, remained above the threshold of 1.0 × 10^−6^. This finding aligns with previous research indicating that Cr has been a principal contributor to the CR of PM_2.5_-bound metals in Jinan. Consequently, the implementation of stringent Cr control measures in the region is imperative [64].

The PMF-based source apportionment revealed that Mn, As, and Cr were predominantly sourced from industrial activities and vehicular emissions. It is therefore suggested that the observed decreases in the HQ of these metals were intimately connected to the efficacy of emission control measures directed at the contributing sources, such as the transformation of industrial processes, the retirement of old vehicles, and the optimization of fuel formulations, etc. Despite the suite of air pollution mitigation strategies implemented during the 13th Five-Year Plan period, the CR of some metals was still higher than the acceptable levels. There is a need for stricter control of carcinogenic metal emissions in the region. Recommended measures include harnessing clean energy sources—wind and solar power—to supplant thermal power generation, the further realization of full public transportation, and the promotion of new energy vehicles. As of 2023, the share of clean energy has increased (about 15%) in the region. Additionally, the local government subsidizes the price of new energy vehicles, the share of new energy vehicles has increased (about 10%), and new subway lines have been constructed, demonstrating the feasibility of these strategies.

#### 3.3.2. Source-Specific Health Risk Apportion Based on the RM-RA Model

**Figure 5:**
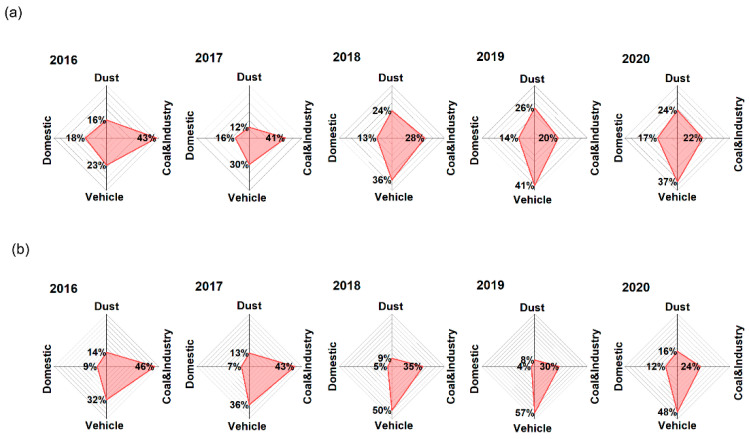
Annual trends in the contributions of emission sources to (**a**) HI; (**b**) TCR.

Assessing health risks attributable to specific sources is essential for identifying and prioritizing the most critical pollution sources for control efforts. The average HI to adults and children from sources between 2016 and 2020 were coal combustion and industrial emissions (0.11 and 0.18), vehicle emissions (0.10 and 0.17), dust emissions (0.07 and 0.11), and domestic emissions (0.05 and 0.09). Of these, HI from coal combustion and industrial emissions had declined significantly by 66% between 2016 and 2020, while there was an upward trend in HI for both dust emissions and vehicle emissions, which peaked in 2019 (declining in 2020 due to the impact of COVID-19) (Figure S6). As shown in Figure 5a, the contribution of coal combustion and industrial emissions to HI decreased from 43% (in 2016) to 22% (in 2020), with a large drop occurring from 2017 to 2018. Jinan had completed the relocation of a large steel plant by the end of 2017; at the same time, the transformation of some heavily polluting industries had also been gradually completed [64]. In contrast, the proportion of the HI attributable to vehicle emissions experienced an increase, escalating from 23% in 2016 to 37% by the year 2020, which can be related to the continued increase in vehicle ownership in Jinan.

During the study period, the sources of the average CR for adults and children included vehicle emissions (6.3 × 10^−6^), coal combustion and industrial emissions (5.4 × 10^−6^), dust emission (1.7 × 10^−6^), and domestic emissions (1.1 × 10^−6^), all of which exceeded the acceptable level (1.0 × 10^−6^) (Figure S6). The CR of all sources in 2020 was lower than in 2016, but their contributions to TCR had changed significantly. As shown in Figure 5b, coal and industrial emissions and vehicle emissions contributed 81% of the TCR, and previous studies have shown that these are two sources of concern [65,66]. Although dust emissions contributed the most to the mass concentration of TMs (about 50%), a separate study found a high proportion of non-carcinogenic metals in dust emissions, especially Al, which is the main reason for its low CR compared with other sources [67]. The contributions of coal combustion and industrial emissions to the TCR were relatively high before 2018, while vehicle emissions had become dominant since 2018, contributing more than 50% of the TCR, with the exception of 2020, due to travel restrictions associated with COVID-19 pandemic control that significantly reduced the use of vehicles [68,69].

Overall, coal combustion and industrial emissions and vehicle emissions were the two sources of greatest concern in Jinan, and alternated in their dominance in contributing to health risks around 2017, which may be related to the upgrading of the energy structure, the modification of industry, and the continuous rise in vehicle ownership in Jinan. Our results advocate for a greater focus on vehicle emissions, such as by promoting electric vehicles, strengthening emission standards, and phasing out older and less efficient vehicles. At the same time, the contributions of dust and domestic emissions to health risks are relatively small, but not negligible, and there is still a need to strengthen controls such as wet operations, road cleaning, and the promotion of a green lifestyle.

### 3.4. Policy Implications

**Figure 6:**
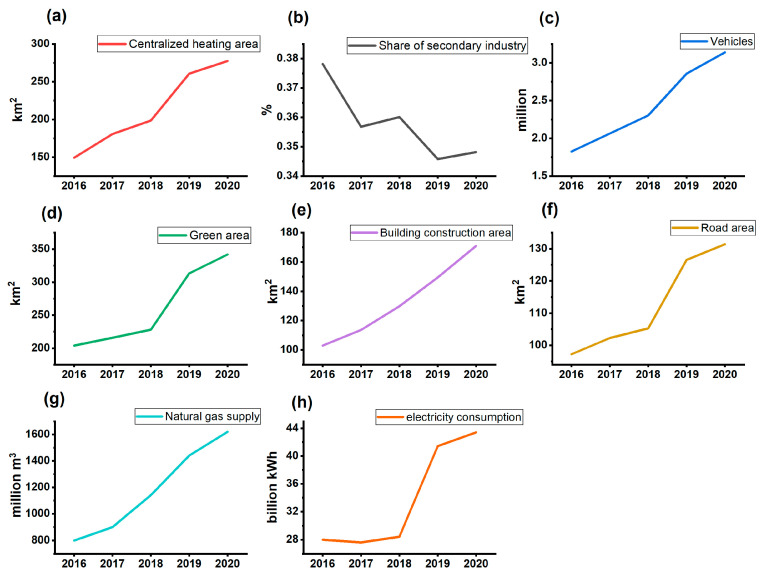
Factors affecting the emission sources: (**a**) centralized heating area; (**b**) share of secondary industry; (**c**) number of vehicles; (**d**) green area; (**e**) building construction area; (**f**) road area; (**g**) natural gas supply; (**h**) electricity consumption. The data are from Jinan Statistical Yearbook (jinan.gov.cn, accessed on 10 July 2024).

During the study period, the HI from all sources was determined to be below the levels deemed acceptable. Concurrently, the CR experienced a decline, yet it persisted above the established acceptable thresholds. As shown in Figure S6, the CR attributable to coal combustion and industrial emissions saw a marked reduction by 67.3% during the study period. The substantial alterations within this period are presumably linked to Jinan’s prioritized efforts in mitigating industrial pollution emissions, established as a critical component of the 13th Five-Year Plan (2016–2020), including the relocation of a large iron and steel plant and the renovation of highly polluting enterprises, such as technological renovations for power and steam, metallurgy, and building material industries with high energy consumption and emissions (Table S6). The centralized heating area in Jinan continued to rise during the period, effectively improving the reduction in coal consumption (Figure 6a). The GDP share of the secondary industry continued to decline between 2016 and 2020, from 37.8% to 34.8% (Figure 6b). These measures contributed to the improvement of air quality and the continuous decrease in emission source concentrations and CR [70].

The CR associated with vehicle emissions fluctuated between 1.0 × 10^−5^ and 4.0 × 10^−6^, surpassing the permissible risk level of 1 × 10^−6^. The CR reduction for vehicle emissions was relatively small (6.8%), as the effects of vehicle emission controls were largely offset by increasing vehicle ownership. From 2016 to 2020, the number of vehicles in Jinan increases by 81%, with the share of new energy vehicles gradually increasing (Figure 6c). Jinan has fully realized the electrification of buses, cabs, net cars, and sanitation vehicles. A deeper analysis had revealed that vehicle non-exhaust emissions (NEE), encompassing activities such as braking and tire abrasion, had become a noteworthy source of health hazards, and their tracer elements contribute highly to the CR of vehicle emissions, such as Cr (72%) and Ni (14%) [71,72]. This is closely related to car ownership (including new energy vehicles) and traffic congestion problems in Jinan. The previous study showed an increase in the total trace element emissions due to braking and tire wear in China, suggesting that NEE matter is increasingly surfacing as a critical environmental and health concern [73,74]. At present, numerous urban areas in China have adopted policies restricting the license plate numbers of motor vehicles to mitigate emissions during peak traffic periods and to address critical air pollution incidents [75]. Furthermore, the extensive proliferation of new energy vehicles in the Pearl River Delta region has resulted in a significant reduction in the levels of elemental carbon and associated metal concentrations [76]. These initiatives provide enlightening guidance for the regulation of PM_2.5_ emissions, particularly those associated with metals, from vehicles in Jinan.

The CR of dust emissions decreased by 26.6% during 2016–2020. Jinan enforced targeted measures for the suppression and management of dust emissions during construction processes, implementing wet work at construction sites since 2016, while continuously increasing the green area of the urban area (Figure 6d), from 204 km^2^ (2016) to 342 km^2^ (2020). Despite ongoing efforts, the escalation of construction projects, including architectural and road infrastructure developments, has introduced considerable difficulties in controlling dust emissions (Figure 6e,f). For instance, the area of building construction has continued to grow from 103 km^2^ (2016) to 171 km^2^ (2020). The CR for domestic emissions is overall a downward trend attributable to increased environmental awareness, such as the use of natural gas and electricity in place of coal (Figure 6g,h). The elevation seen in 2020 was influenced by COVID-19 during that time period, where residents were required to stay in their homes, inevitably increasing indoor metal emissions [77].

## 4. Conclusions

This study comprehensively investigated the sources of PM_2.5_-bound metals and their health risks in Jinan from 2016 to 2020. The results indicated that the concentrations of most metals decreased over the study period, while a significant seasonal difference was observed due to the interplay between meteorological factors and human activities, with the highest concentrations in winter. PMF source analysis categorized the metals into four sources, whose contributions to the concentrations of the TMs were dust emissions > coal combustion and industrial emissions > vehicle emissions > domestic emissions, and which dynamically changed under the influence of emission reduction policies. The risk assessment outcomes indicated that the HQ of all metals was minimal, while the CRs posed by certain metals, specifically Cr and As, warrant significant concern. Combined with the PMF model, coal combustion and industrial emissions and vehicle emissions are the two main sources of hazard. Emission reduction policies have played a role in reducing the health risks of pollution sources. However, with the continuous growth in the number of vehicles and the frequent occurrence of traffic congestion, reducing vehicle emissions, especially NEEs, will be a new challenge. 

In the 14th Five-Year Plan era (2021–2025), set against the backdrop of China’s swift urbanization, the government has enhanced its efforts to curb emissions across key sectors including industry, energy, transportation, and dust pollution. Specifically, the measures entail an aggressive reduction of industries with high energy consumption, a robust expansion of clean energy sources, a substantial enhancement of the green transportation infrastructure, and an in-depth intensification of dust pollution control strategies. These emission reduction measures will have a positive impact on reducing the concentration of PM_2.5_ and its constituents (www.gov.cn, accessed on 12 July 2024).

## Data Availability

The observational data obtained in this study are available from the corresponding authors upon request (202236505@mail.sdu.edu.cn).

## Supplementary Materials

The following supporting information can be downloaded at: https://www.mdpi.com/article/10.3390/toxics12080581/s1, Section S1: PMF analysis; Figure S1: Trends in metal concentrations in the Shizhong District (SZ) and Licheng District (LC) during the study period; Figure S2: Correlation between the same metals in the two monitoring regions; Figure S3: Correlation analysis between pollutants and meteorological parameters in Jinan between 2016 and 2020. Relative humidity (RH); temperature (T); atmospheric pressure (AP); wind speed (WS); total metals (TMs); Figure S4: Comparison between different factors obtained by PMF; Figure S5: PMF analysis results: (a) profiles of four factors for PM_2.5_-bound metals, (b) contributions of four possible sources in PM_2.5_-bound metals, (c) contribution of different metals to the sources; Figure S6: Annual trends in health risks from emission sources: (a) non-carcinogenic risks (darker colors indicate adults, lighter colors indicate children); (b) carcinogenic risks (gray straight lines indicate acceptable levels of 1 × 10^−6^); Table S1: Exposure parameters used for health risk assessment (referring to Chinese Exposure Factors Handbook); Table S2: Exposure dose–response parameters and date sources; Table S3: Summary statistics for PM_2.5_ mass and its metal element mass concentrations in Jinan between 2016 and 2020 (all values are in units of ng/m^3^); Table S4: Annual trends in HQ of different metals for adults and children in Jinan between 2016 and 2020; Table S5: Annual trends in CR of different metals for adults and children in Jinan between 2016 and 2020; Table S6: Major PM_2.5_ emission reduction policies in Jinan during the 13th Five-Year Plan period (2016–2020). References [78,79,80,81,82] are cited in the Supplementary File.
