# Supplementary material for: Sources and Specified Health Risks of 12 PM2.5-Bound Metals in a Typical Air-Polluted City in Northern China during the 13th Five-Year Plan
Source: Toxics. 2024 Aug 10;12(8):581. doi: 10.3390/toxics12080581 (PMC11360060; doi:10.3390/toxics12080581)
Supplement: Supplementary file 1 [file toxics-12-00581-s001.zip › toxics-3131020-supplementary.pdf]

PMF analysis

Positive Matrix Factorization (PMF) requires the uncertainty of chemical species as input data. The uncertainty (UNC) calculation method is as follows.

(1) If the concentration is lower than the minimum detection limit (MDL),

$$UNC = \frac{5}{6} \times MDL;$$

(2) If the concentration is higher than the MDL,

$$UNC = \sqrt{(error\ rate \times mass\ concentration)^2 + (MDL)^2}$$

The concentrations of Hg elements exceeding 50% are lower than the detection limit and are not input into the model. In addition, the total element concentrations of Sb, Al, As, Be, Cd, Cr, Pb, Mn, Ni, Se, and Tl are set as the total variables, and the uncertainties of metals are 5%. PMF version 5.0 was used in this work. In this study, six factors in the PMF model were tested (Figure S3). Among three factors, Factor 1 is mainly composed of As, Cd, Cr, Pb, Ni, Se, and Tl. Pb, Cd, and Se are industrial source tracers, while Mn, Ni, and Cr are vehicle source tracers. Therefore, Factor 1 is clearly not separated. Under the condition of three factors, the correlation between the observation and simulation of most elements is approximately 0.5, which is poor, and $Q/Qexp$ is 1.8. The condition of four factors is discussed in the results section of the main text. The corresponding $Q/Qexp$ is 1.5, and the simulation of the model has a good fit (R^2 = 0.92, slope = 0.95) with the observations. When the number of factors exceeds four, the vehicle and industrial sources will be decomposed into multiple sources, and the difference between the simulated value and the observed value of the component will become larger. For example, among five factors, we can conclude that the Cr element is simulated as a factor, although Cr is usually considered for tire wear, brake pads, and in some regions found in the metallurgical industry.

In order to test the stability of the optimal solution, error estimation analysis was employed on the four-factor solution, including bootstrap (BS), displacement (DISP), and bootstrap with displacement (BS-DISP). Given that sufficient BS runs can provide the statistical uncertainty of the PMF solutions, in this work, 100 BS runs of 88 were performed for the PMF solution and an r value of 0.8 was set to map bootstrapped and base case factor contributions. All species (Sb, Al, As, Be, Cd, Cr, Pb, Mn, Ni, Se, and Tl) in each factor profile were selected to displace in the BS-DISP analysis. All factors were mapped for 100% of BS runs, no swapping in DISP or BS-DISP occurred,

and 100% of cases accepted in BS-DISP were observed, indicating that the solution with four factors was appropriate and sufficiently robust.

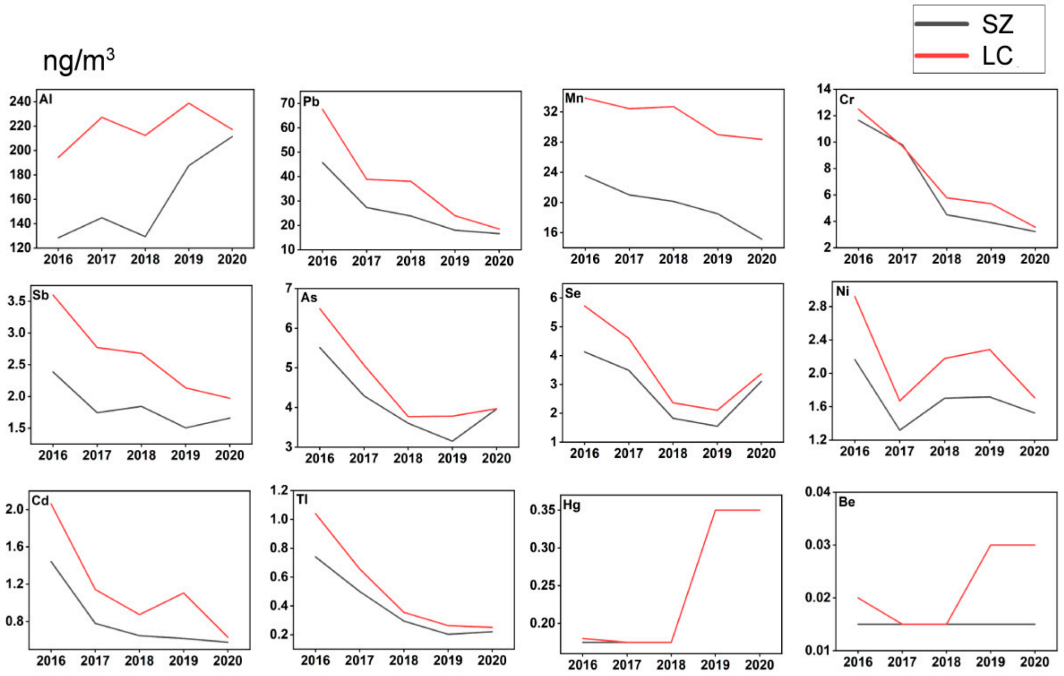

Figure S1. Trends in metal concentrations in the Shizhong District (SZ) and Licheng District (LC) during the study period.

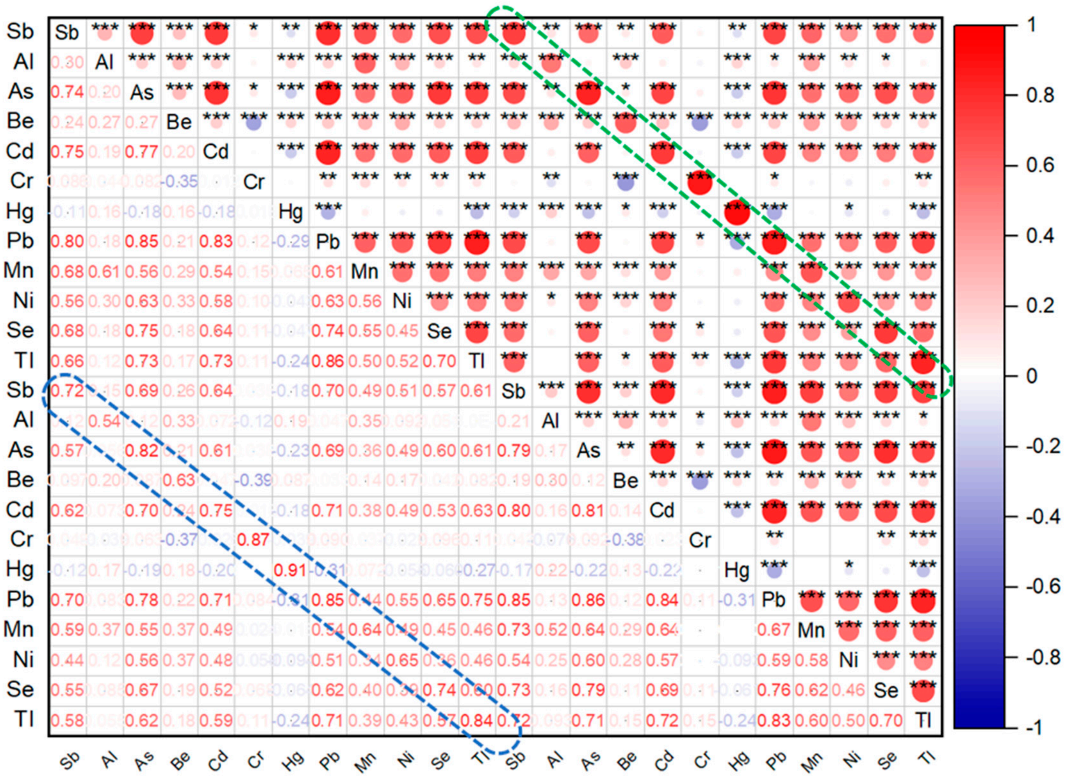

Figure S2. Correlation between the same metals in the two monitoring regions.

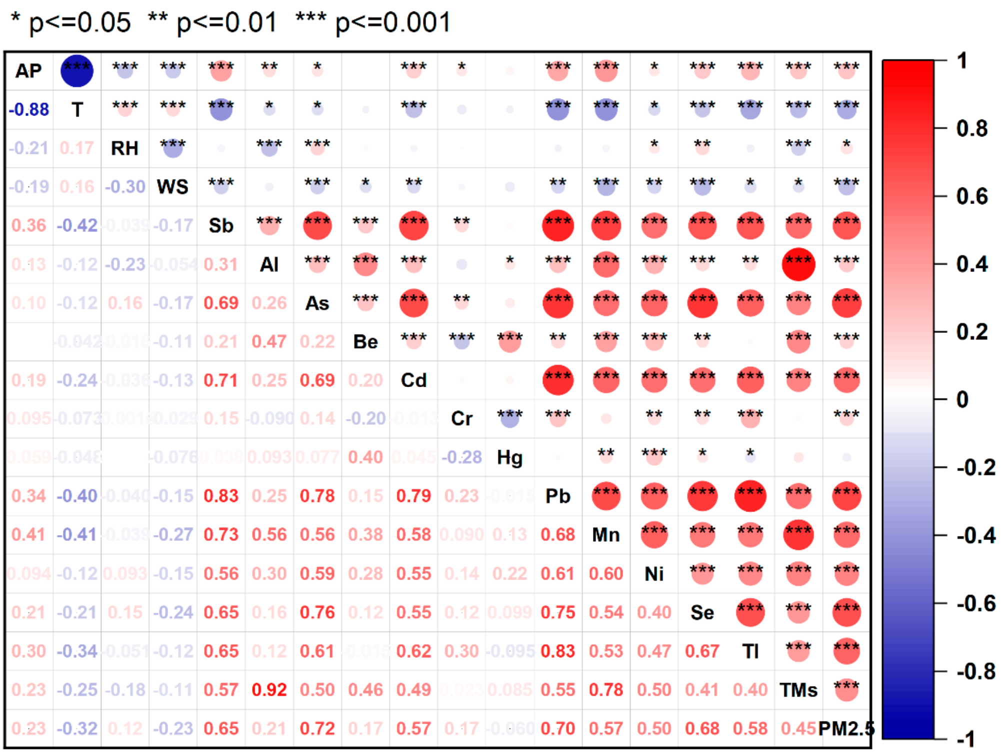

Figure S3. Correlation analysis between pollutants and meteorological parameters in Jinan between 2016 and 2020. Relative humidity (RH); temperature (T); atmospheric pressure (AP); wind speed (WS); total metals (TMs).

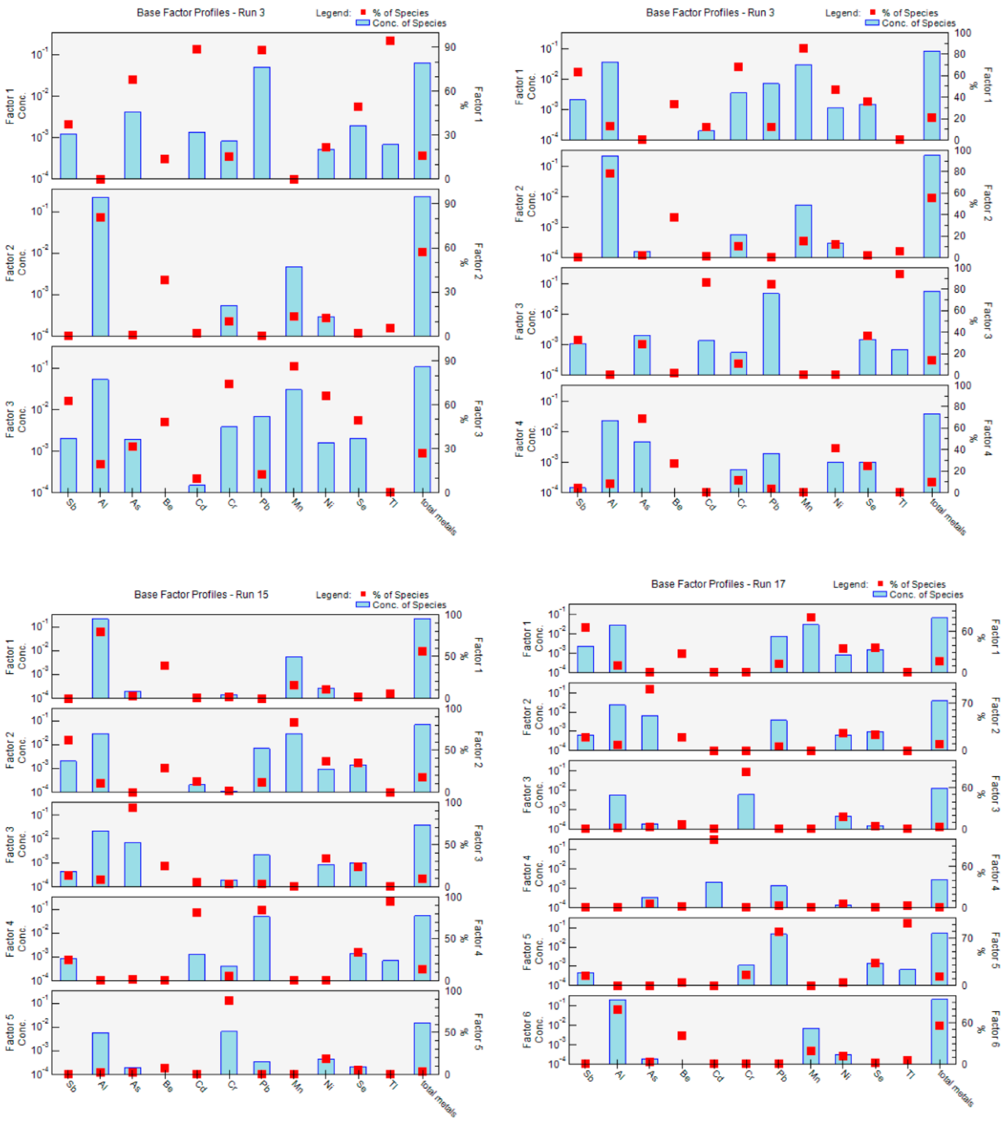

Figure S4. Comparison between different factors obtained by PMF.

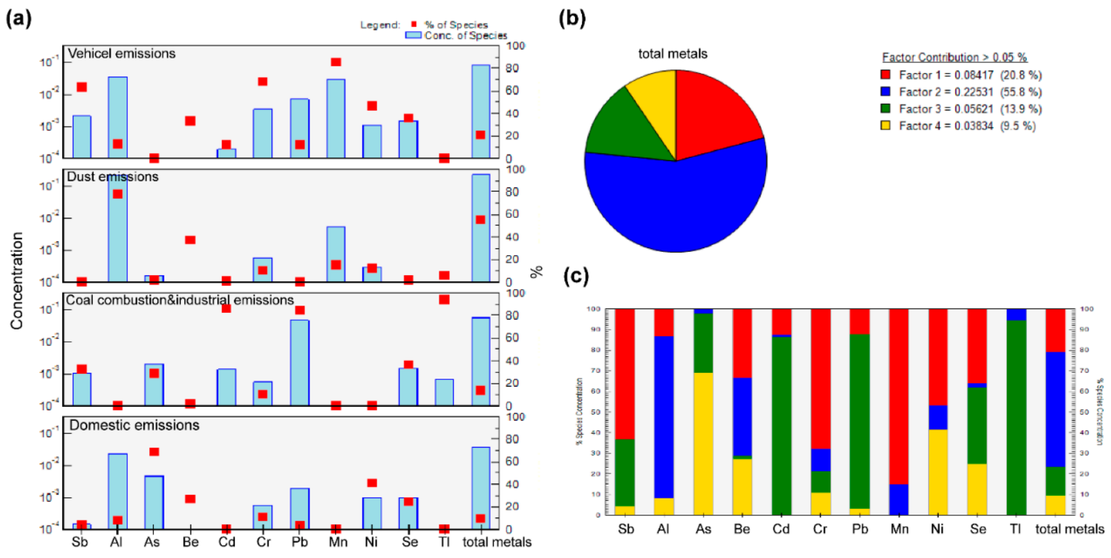

Figure S5. PMF analysis results: (a) profiles of four factors for $PM_{2.5}$-bound metals, (b) contributions of four possible sources in $PM_{2.5}$-bound metals, (c) contribution of different metals to the sources.

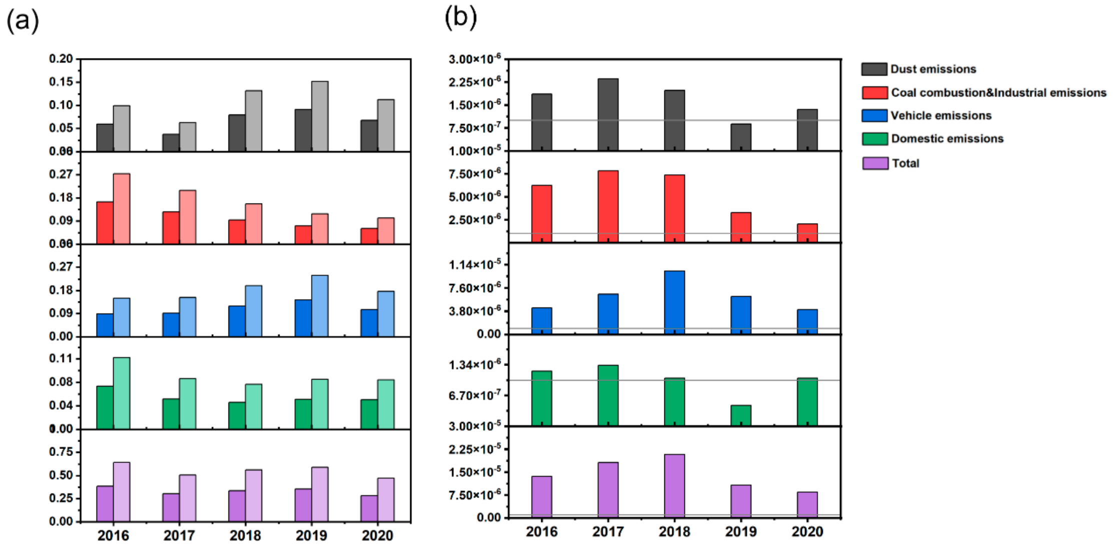

Figure S6. Annual trends in health risks from emission sources: (a) non-carcinogenic risks (darker colors indicate adults, lighter colors indicate children); (b) carcinogenic risks (gray straight lines indicate acceptable levels of 1×10^{-6}).

Table S1. Exposure parameters used for health risk assessment (referring to Chinese Exposure Factors Handbook [78]).

Parameter	Distribution	Unit
Exposure duration (ED)	30 (adult); 6 (children)	years
Averaging time (AT)	ED×365	days
Lifetime (LT)	76×365	days
Exposure frequency (EF)	365	days
Body weight (BW)	65 (adult); 22 (children)	kg
Inhalation rate (IR)	16.1 (adult); 9 (children)	m^3/day

Table S2. Exposure dose–response parameters and date sources.

Element	Non-carcinogenic		Carcinogenic	
	RfC (mg/m^3)	Date source	IUR [$(ug/m^3)^{-1}$]	Date source
Al	5.0×10^{-3}	PPRTVs [79]		
Hg	3.0×10^{-4}	IRIS [80]		
Mn	5.0×10^{-5}	IRIS [80]		
Sb	3.0×10^{-4}	ATSDR [82]		
Se	1.0×10^{-3}	CALEPA [81]		
As	1.5×10^{-5}	CALEPA [81]	4.3×10^{-3}	IRIS [80]
Be	2.0×10^{-5}	IRIS [80]	2.4×10^{-3}	IRIS [80]
Cr	1.0×10^{-4}	IRIS [80]	1.2×10^{-2}	IRIS [80]
Cd	1.0×10^{-5}	ATSDR [82]	1.8×10^{-3}	IRIS [80]
Ni	9.0×10^{-5}	ATSDR [82]	2.4×10^{-4}	IRIS[80]
Pb			1.2×10^{-5}	CALEPA[81]

Table S3. Summary statistics for $PM_{2.5}$ mass and its metal element mass concentrations in Jinan between 2016 and 2020 (all values are in units of ng/m^3).

Pollutants (n=442)	Mean	SD	Min	Median	Max
Sb	3.2E+00	2.8E+00	3.1E-01	2.4E+00	2.8E+01
Al	2.3E+02	1.4E+02	5.4E+01	2.0E+02	1.2E+03
As	5.2E+00	3.7E+00	5.7E-01	4.3E+00	2.8E+01
Be	2.6E-02	2.3E-02	1.1E-02	2.0E-02	2.5E-01
Cd	1.7E+00	2.1E+00	1.2E-02	9.1E-01	1.3E+01
Cr	7.3E+00	4.9E+00	2.5E-01	5.7E+00	2.7E+01
Hg	2.0E-01	1.3E-01	2.5E-03	1.8E-01	9.3E-01
Pb	4.4E+01	3.9E+01	4.6E+00	3.0E+01	2.5E+02
Mn	3.1E+01	1.9E+01	4.7E+00	2.6E+01	1.2E+02
Ni	2.3E+00	1.4E+00	5.3E-01	2.0E+00	1.6E+01
Se	3.7E+00	2.6E+00	4.7E-01	3.0E+00	1.7E+01
Tl	6.3E-01	6.1E-01	4.5E-02	3.6E-01	3.2E+00
TMs	3.3E+02	1.7E+02	8.3E+01	2.9E+02	1.6E+03
$PM_{2.5}$	6.6E+04	3.8E+04	7.5E+03	5.6E+04	2.5E+05

Table. S4. Annual trends in HQ of different metals for adults and children in Jinan between 2016 and 2020.

Sorts	Metals	2016		2017		2018		2019		2020	
		Mean	SD	Mean	SD	Mean	SD	Mean	SD	Mean	SD
Adult											
	Mn	1.7E-01	8.3E-02	1.4E-01	9.8E-02	1.8E-01	1.4E-01	2.0E-01	1.4E-01	1.6E-01	1.1E-01
	As	1.2E-01	8.5E-02	8.7E-02	6.0E-02	7.0E-02	4.1E-02	6.8E-02	4.1E-02	7.2E-02	4.9E-02
	Cd	6.3E-02	5.7E-02	3.6E-02	4.0E-02	3.5E-02	4.7E-02	5.3E-02	8.3E-02	1.9E-02	1.4E-02
	Al	1.1E-02	1.1E-02	1.2E-02	9.0E-03	1.2E-02	7.6E-03	1.3E-02	9.4E-03	1.1E-02	5.4E-03
	Be	3.5E-04	5.6E-04	2.6E-04	1.9E-04	2.2E-04	1.2E-04	3.8E-04	1.9E-04	4.5E-04	3.9E-04
	Cr	1.4E-02	4.6E-03	2.3E-02	1.5E-02	2.8E-02	1.3E-02	1.2E-02	8.8E-03	9.0E-03	5.0E-03
	Hg	1.4E-04	1.0E-04	1.3E-04	0.0E+00	7.2E-05	6.2E-05	2.3E-04	5.9E-05	1.6E-04	1.2E-04
	Sb	3.3E-03	2.3E-03	2.6E-03	2.0E-03	3.0E-03	3.1E-03	2.7E-03	3.1E-03	2.1E-03	1.6E-03
	Ni	8.5E-03	4.9E-03	4.3E-03	2.3E-03	6.9E-03	4.2E-03	6.8E-03	4.5E-03	5.2E-03	4.0E-03
	Se	1.4E-03	6.6E-04	1.2E-03	6.9E-04	6.3E-04	4.7E-04	5.4E-04	3.2E-04	9.1E-04	5.4E-04
	SUM	3.9E-01	1.9E-01	3.1E-01	1.8E-01	3.4E-01	2.2E-01	3.6E-01	2.0E-01	2.8E-01	1.7E-01
Child											
	Mn	2.7E-01	1.4E-01	2.3E-01	1.6E-01	3.0E-01	2.3E-01	3.3E-01	2.3E-01	2.7E-01	1.9E-01
	As	2.0E-01	1.4E-01	1.4E-01	9.9E-02	1.2E-01	6.7E-02	1.1E-01	6.8E-02	1.2E-01	8.2E-02
	Cd	1.0E-01	9.4E-02	5.9E-02	6.7E-02	5.8E-02	7.7E-02	8.7E-02	1.4E-01	3.1E-02	2.3E-02
	Al	1.7E-02	1.8E-02	2.0E-02	1.5E-02	2.0E-02	1.3E-02	2.2E-02	1.5E-02	1.9E-02	8.9E-03
	Be	5.8E-04	9.3E-04	4.3E-04	3.2E-04	3.7E-04	2.0E-04	6.2E-04	3.1E-04	7.4E-04	6.4E-04
	Cr	2.3E-02	7.5E-03	3.8E-02	2.4E-02	4.7E-02	2.2E-02	2.0E-02	1.4E-02	1.5E-02	8.3E-03
	Hg	2.4E-04	1.7E-04	2.2E-04	0.0E+00	1.2E-04	1.0E-04	3.8E-04	9.6E-05	2.6E-04	1.9E-04
	Sb	5.4E-03	3.7E-03	4.2E-03	3.3E-03	4.9E-03	5.0E-03	4.4E-03	5.2E-03	3.5E-03	2.6E-03
	Ni	1.4E-02	8.1E-03	7.0E-03	3.7E-03	1.1E-02	7.0E-03	1.1E-02	7.4E-03	8.7E-03	6.6E-03
	Se	2.2E-03	1.1E-03	2.0E-03	1.1E-03	1.0E-03	7.7E-04	8.9E-04	5.3E-04	1.5E-03	8.9E-04
	SUM	6.4E-01	3.1E-01	5.1E-01	3.0E-01	5.6E-01	3.6E-01	5.9E-01	3.3E-01	4.7E-01	2.8E-01

Table. S5. Annual trends in CR of different metals for adults and children in Jinan between 2016 and 2020.

Metals	2016		2017		2018		2019		2020	
	Mean	SD	Mean	SD	Mean	SD	Mean	SD	Mean	SD
Cr	8.63E-06	2.89E-06	1.47E-05	9.27E-06	1.80E-05	8.51E-06	7.85E-06	5.57E-06	5.71E-06	3.20E-06
As	4.10E-06	2.89E-06	2.97E-06	2.05E-06	2.39E-06	1.39E-06	2.30E-06	1.40E-06	2.47E-06	1.68E-06
Cd	5.99E-07	5.37E-07	3.38E-07	3.83E-07	3.34E-07	4.46E-07	5.04E-07	7.89E-07	1.76E-07	1.31E-07
Pb	1.22E-07	7.70E-08	7.29E-08	6.07E-08	6.66E-08	5.45E-08	4.67E-08	3.48E-08	4.60E-08	4.34E-08
Ni	9.72E-08	5.63E-08	4.87E-08	2.58E-08	7.88E-08	4.84E-08	7.77E-08	5.13E-08	5.99E-08	4.55E-08
Be	8.84E-09	1.42E-08	6.57E-09	4.88E-09	5.68E-09	3.07E-09	9.54E-09	4.71E-09	1.13E-08	9.81E-09
SUM	1.36E-05	4.69E-06	1.81E-05	9.18E-06	2.09E-05	9.18E-06	1.08E-05	5.83E-06	8.46E-06	4.59E-06

Table S6. Major $PM_{2.5}$ emission reduction policies in Jinan during the 13th Five-Year Plan period (2016-2020).

Year	Policy documents
2016-2017	***Shandong Province's 2013-2020 Air Pollution Prevention and Control Plan Phase II Action Plan (2016-2017)***
2016.10	Jinan City Air Pollution Prevention and Control Action Plan (Phase II)
2017.01	Notice of the General Office of the People's Government of Jinan City on Issuing Several Measures for the Control of Dust Pollution in Construction Projects in Jinan City
2017.03	Notice on the Implementation Plan for the Promotion of Clean Civil Domestic Coal Combustion in Jinan City
2017.09	Notice on the Implementation Plan of Dust Control Action in Autumn and Winter of Jinan City from 2017 to 2018
2017.10	Notice on the Three Year Implementation Plan for the "Winter Clean Heating Pilot City in the Northern Region" of Jinan City (2017-2020)
2017.12	Notice on the Comprehensive Work Plan for Energy Conservation and Emission Reduction during the 13th Five Year Plan period in Jinan City
2017.12	Notice of the People's Government of Jinan City on Prohibiting the Passage of Heavy Duty Diesel Trucks that Do Not Meet the National IV Emission Standards
2018-2020	***The Operational Plan for Winning the Blue Sky Defense War in Shandong Province and the Phase III Action Plan for Air Pollution Prevention and Control Plan 2013-2020 (2018-2020)***
2018.12	Jinan City Triennial Action Plan for Winning the Blue Sky Defense War and Air Pollution Prevention and Control Action Plan (Phase III)
2018.01	Implementation Plan for Promoting High Quality Civil Coal Combustion during the Heating Season of Jinan City from 2018 to 2019
2018.04	Implementation Plan for Subsidies for Scrapping and Updating Old Diesel Vehicles in Jinan City
2019.01	Notice of Jinan Municipal People's Government on Delineating Low Emission Control Zones for Non road Mobile Machinery
2019.12	Notice of the People's Government of Jinan City on Delineating High Emission Prohibited Zones and Low Emission Control Zones for Non road Mobile Machinery
2020.07	Notice of Jinan City on Prohibiting the Passage of Diesel Trucks Below the National IV Emission Standards

References

78. Wang, B.; Cao, S.; Zhao, X.; Dong, T.; Nie, J.; Duan, X. Time-Activity Factors Related to Air Exposure. In Highlights of the Chinese Exposure Factors Handbook; Duan, X., Zhao, X., Wang, B., Chen, Y., Cao, S., Eds.; Academic Press: Beijing, China, 2015; pp. 31–39 ISBN 978-0-12-803125-4.
79. The Provisional Peer Reviewed Toxicity Values [DB/OL]. (2017). [cited 2024 August 5]; Available from: https://hhpprtv.ornl.gov/quickview/pprtv.php.
80. IRIS. (2017). IRIS advanced search [DB/OL]. [cited 2024 August 5]; Available from: https://cfpub.epa.gov/ncea/iris2/atoz.cfm.
81. CALEPA. (2017). OEHHA chemical database—Air [DB/OL]. [cited 2024 August 5]; Available from: https://oehha.ca.gov/air/chemicals.
82. Minimal Risk Levels for Hazardous Substances (MRLs) [DB/OL]. (2017). [cited 2024 August 5]; Available from: https://www.atsdr.cdc.gov/mrls/mrllist.asp#39tag.
